# Palmitoylated Proteins in Dendritic Spine Remodeling

**DOI:** 10.3389/fnsyn.2020.00022

**Published:** 2020-06-16

**Authors:** Joseph P. Albanesi, Barbara Barylko, George N. DeMartino, David M. Jameson

**Affiliations:** ^1^Department of Pharmacology, University of Texas Southwestern Medical Center, Dallas, TX, United States; ^2^Department of Physiology, University of Texas Southwestern Medical Center, Dallas, TX, United States; ^3^Department of Cell and Molecular Biology, University of Hawaii, Honolulu, HI, United States

**Keywords:** palmitoylation, dendritic spines, cytoskeletal remodeling, actin assembly, synaptic plasticity

## Abstract

Activity-responsive changes in the actin cytoskeleton are required for the biogenesis, motility, and remodeling of dendritic spines. These changes are governed by proteins that regulate the polymerization, depolymerization, bundling, and branching of actin filaments. Thus, processes that have been extensively characterized in the context of non-neuronal cell shape change and migration are also critical for learning and memory. In this review article, we highlight actin regulatory proteins that associate, at least transiently, with the dendritic plasma membrane. All of these proteins have been shown, either in directed studies or in high—throughput screens, to undergo palmitoylation, a potentially reversible, and stimulus-dependent cysteine modification. Palmitoylation increases the affinity of peripheral proteins for the membrane bilayer and contributes to their subcellular localization and recruitment to cholesterol-rich membrane microdomains.

## Introduction

Protein palmitoylation is a key regulatory mechanism in synaptic plasticity (recently reviewed by Globa and Bamji, 2017; Zaręba-Kozioł et al., 2018; Matt et al., 2019). The majority of studies examining the role of palmitoylation in synapse remodeling have focused on the modification of proteins involved in regulating the activity, localization, and trafficking of synaptic receptors, primarily AMPA-type glutamate receptors (AMPARs). However, sustained changes in synaptic efficacy, which are underlying mechanisms of learning and memory, are also determined by changes in the number and morphology of dendritic spines (Cingolani and Goda, 2008; Kasai et al., 2010) and, hence, by activity-dependent changes in the actin cytoskeleton. Several regulators of actin assembly in dendrites are peripheral membrane proteins that undergo palmitoylation, the covalent modification of cysteines with 16-carbon palmitoyl chains. In addition to increasing a protein’s affinity for membranes by the hydrophobicity of the palmitoyl moiety, palmitoylation can influence protein stability, subcellular localization, and protein-protein interactions. Moreover, unlike myristoylation and prenylation, palmitoylation is reversible and may respond to changes in neuronal conditions.

Here, we present an overview of palmitoylated proteins that regulate actin polymerization in neurons. Peripheral membrane proteins discussed in this review include two Rho family GTPases (Rac1 and Cdc42), a downstream kinase activated by Rho GTPases (LIMK1), and two proteins that directly participate in both endocytic membrane trafficking and actin polymerization (Arc and PICK1). We also discuss two palmitoylated integral membrane proteins, tetraspanin 7 (TSPAN7) and tetraspanin 5 (TSPAN5), which have been implicated in spine remodeling. Palmitoylation has been established as a regulatory mechanism for the actin remodeling activity of only three of these proteins, Cdc42, Rac1, and LIMK1. Further studies will be needed to determine how (or if) palmitoylation influences the activities of PICK1, Arc, and the tetraspanins. Numerous recent reviews discuss more generally the role of actin dynamics in dendritic spine remodeling (for example, Frost et al., 2010; Spence and Soderling, 2015; Chazeau and Giannone, 2016; Hlushchenko et al., 2016; Lei et al., 2016; Bertling and Hotulainen, 2017; Fu and Ip, 2017; Konietzny et al., 2017; Borovac et al., 2018; Newpher et al., 2018).

## Role of Palmitoylation in Targeting Actin Regulatory Proteins to Membrane Subdomains

Cholesterol binds preferentially to saturated fatty acids, such as myristate and palmitate, over unsaturated fatty acids (Radhakrishnan et al., 2000; Ali et al., 2007). This observation is commonly invoked to explain the partitioning of acylated proteins to lipid rafts, which are operationally defined as small (10–200 nm) membrane microdomains, enriched in cholesterol, sphingolipids, and saturated phospholipids, serving to compartmentalize cellular processes (Pike, 2006). Based on experiments on isolated plasma membranes, the contribution of palmitoyl chains to raft partitioning free energy (ΔG_palm_) has been estimated as −0.48 kcal/mol per acyl chain (Lorent et al., 2017). With only 14 carbons, myristoyl chains alone are insufficient to target proteins to rafts, but they contribute to raft affinity in the many examples of dually myristoylated and palmitoylated proteins (Levental et al., 2010; Lorent and Levental, 2015). In contrast, branched and unsaturated prenyl groups (farnesyl or geranylgeranyl) favor non-raft membranes (Melkonian et al., 1999; Levental et al., 2010). Consistent with the abundance of palmitoylated proteins in postsynaptic densities (PSDs), the postsynaptic plasma membrane (PM) is believed to be highly enriched in lipid rafts (Suzuki, 2002; Suzuki et al., 2011). Indeed, the PSD can be viewed as a phase-separated assembly (Chen et al., 2020) that clusters palmitoylated proteins (particularly PSD95) at high concentrations, leading to the novel proposal that PSD proteins are not only recruited to lipid rafts but participate in their induction and stabilization (Tulodziecka et al., 2016).

Before the advent of single-molecule and super-resolution imaging approaches, proteins were assigned to lipid rafts based largely on their association with detergent-resistant membrane fractions (DRMs), which were found to be enriched in cholesterol, sphingolipids, and glycosylphosphatidylinositol (GPI)-anchored proteins (Brown and Rose, 1992; Schroeder et al., 1994). DRMs are typically separated from non-raft membranes by their insolubility in cold (4°C) non-ionic detergents, often Triton X-100, and their ability to float in density gradients. Numerous postsynaptic proteins have been identified in DRMs, and many of these are palmitoylated (Hering et al., 2003; Zhao et al., 2014). However, the strict equivalence between DRMs and authentic nanodomains in cell membranes has been questioned (Lichtenberg et al., 2005) and has even contributed to skepticism regarding the validity of the lipid raft model (Munro, 2003; Klotzsch and Schütz, 2012; Sevcsik and Schütz, 2016). For example, it was shown that DRMs prepared with different detergents and under different conditions have distinct protein compositions (Schuck et al., 2003; Shogomori and Brown, 2003) and that cold Triton X-100 may even induce the formation of ordered domains (Heerklotz, 2002) or increase domain size (Pathak and London, 2011). Nevertheless, the overwhelming evidence that palmitoylated proteins are enriched in DRMs and are displaced from DRMs upon depalmitoylation reflects a physico-chemical property conferred upon those proteins by acylation.

Cholesterol depletion is another standard criterion to assess whether a protein associates with rafts or if a cellular process is raft-based. Typically, cholesterol levels are reduced by the addition of the sequestering agent methyl-β-cyclodextrin or, less commonly, by inhibition of cholesterol synthesis. Using both procedures, Hering et al. (2003) found that cholesterol depletion reduces spine density, alters spine and overall dendrite morphology, and disrupts the dendritic actin cytoskeleton. Importantly, these effects were reversed by treatment with the actin stabilizing agent, jasplakinolide. Based on these results, the authors concluded that lipid rafts are essential for the maintenance of synapses and dendritic spines, in part due to the dependence of the actin cytoskeleton on raft integrity. However, it must be noted that cholesterol plays a vast role in the regulation of membranes and membrane proteins, most of which may be unrelated to the raft.

Raft organization and function are often dependent on the physical association of rafts with the cortical actin cytoskeleton (Chichili and Rodgers, 2009). The activities of dozens of actin regulatory proteins are controlled by phosphoinositide lipids, particularly phosphatidylinositol 4,5 bisphosphate (PIP_2_) and phosphatidylinositol 3,4,5 triphosphate (PIP_3_; Janmey and Lindberg, 2004; Saarikangas et al., 2010). For example, Rho family GTPases are targeted to the PM both by C-terminal lipid anchors and by electrostatic interactions of their adjoining polybasic regions with PIP_2_ and or PIP_3_ (Lam and Hordijk, 2013; see Figure 2). Early studies on DRMs pointed to the enrichment of phosphoinositides in rafts (Hope and Pike, 1996) and cholesterol depletion experiments suggested a functional significance to this enrichment, particularly concerning cytoskeleton organization (Kwik et al., 2003). At present, it is not entirely clear how raft targeting affects the ability of proteins to access PIP_2_ and PIP_3_ on membranes, as there is disagreement regarding the relative distribution of phosphoinositides between raft and non-raft domains. Phosphoinositides are largely polyunsaturated, with greater than 70% containing one stearoyl chain (C18:0) and one arachidonoyl chain (C20:4; Barneda et al., 2019). Hence, they would be expected to segregate into liquid-disordered non-raft domains. Moreover, the view that PIP_2_ is enriched in rafts domains has been challenged by studies showing that detergent procedures used to prepare DRMs actually induce PIP_2_ condensation and incorporation into DRM fractions (van Rheenen et al., 2005). In addition, super-resolution approaches have revealed the presence of PIP_2_/protein electrostatic clusters that are resistant to cholesterol depletion (van den Bogaart et al., 2011). Current data are insufficient to establish whether palmitoylation localizes actin regulatory proteins in the vicinity of phosphoinositides on the PM, or whether phosphoinositides cluster on rafts due to the concentrated presence in rafts of their palmitoylated effectors.

**Figure 1:**
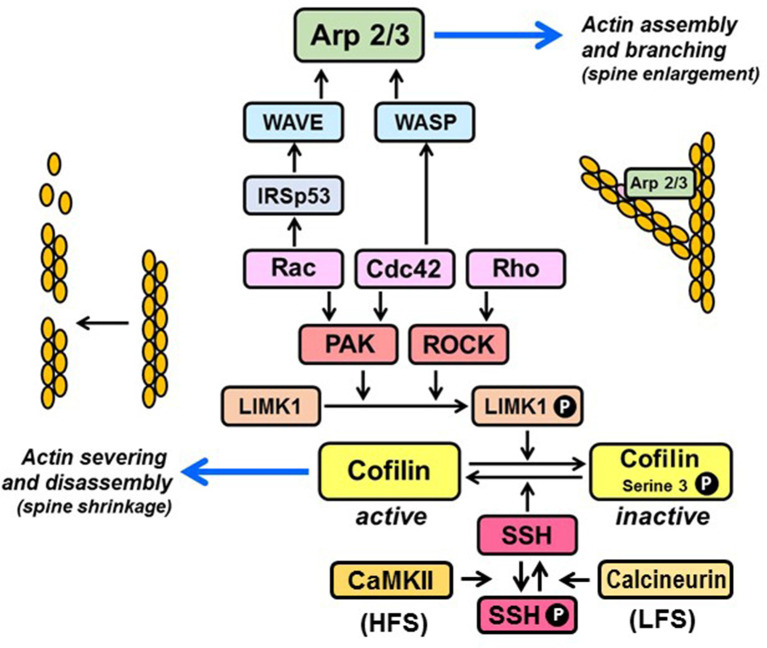
Regulation of actin organization by Rho family GTPases. Rac and Cdc42 promote F-actin polymerization *via* stimulation of p21-activated kinases (PAKs), which phosphorylate and activate LIM kinase-1 (LIMK1). Rho activates LIMK1 by stimulating its downstream kinase ROCK, which phosphorylates LIMK1 directly. Active LIMK1 inhibits cofilin-mediated severing of actin filaments by phosphorylating cofilin on serine-3. Cofilin is re-activated upon dephosphorylation of serine-3 by the phosphatase, slingshot (SSH). SSH is inactivated by CaMKII-mediated phosphorylation, triggered by high-frequency stimulation (HFS) and re-activated upon dephosphorylation by calcineurin, triggered by low-frequency stimulation (LFS). Rac and Cdc42 promote actin branching by activating the nucleation promoting complexes WAVE and WASP, which stimulate the actin filament nucleating activity of the Arp2/3 complex. Rac activation of WAVE is facilitated by binding to the adaptor, IRSp53.

**Figure 2:**
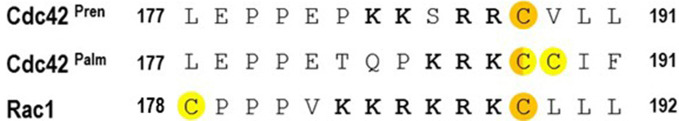
C-terminal sequences of Cdc42 variants and Rac1. Prenylated cysteines are highlighted in orange; palmitoylated cysteines are highlighted in yellow. Note that C188 in Cdc42^Palm^ can undergo both prenylation and palmitoylation. Polybasic motifs are in bold.

In summary, there is little doubt that a major function of palmitoylation is to target proteins to membrane subdomains, whether or not we choose to name them rafts. While the literature on palmitoylation of actin regulatory proteins remains sparse, the role of these proteins in determining spine morphology is well established, as discussed below.

## Changes in The Morphology of Dendritic Spines

### Spine Initiation

Dendritic spines are believed to originate from filopodia (Ziv and Smith, 1996), which are thin, highly dynamic cellular protrusions. Filopodia are particularly motile during synaptogenesis, suggesting that they serve as environmental sensors that extend and retract until they contact their presynaptic partners (Arstikaitis et al., 2008). The filopodia themselves initiate by localized PM bending induced by the I-BAR protein MIM (Missing-In-Metastasis) and then grow as actin filaments assemble beneath these outwardly curved membrane buds (Saarikangas et al., 2015). Most filopodia, such as those found at the periphery of fibroblasts or nerve growth cones, form by extension of unidirectional, unbranched actin filaments, with 15–20 tightly bundled filaments within each filopodium (Le Clainche and Carlier, 2008). In contrast, actin filaments in filopodial dendritic spines are of mixed polarity and undergo at least some level of branching (Hotulainen et al., 2009; Korobova and Svitkina, 2010). In both cases, however, the linear extension is driven by the formin family member mDia2 (Hotulainen et al., 2009).

### Spine Enlargement and Maturation

Synaptic strengthening, as occurs in response to stimuli, such as high-frequency stimulation (HFS) that induce long-term potentiation (LTP), is often accompanied by increases in spine density and volume (Matsuzaki et al., 2004; Okamoto et al., 2004). Thus, filopodial or thin spines, which have also been termed “learning spines,” mature into mushroom-shaped or stubby “memory spines.” These large-headed spines contain high levels of AMPARs, commensurate with their large PSDs. The presynaptic terminals that contact large-headed spines are also larger and contain more synaptic vesicles than those contacting small-headed spines (Kasai et al., 2003). Large-headed spines contain a meshwork of branched actin filaments. As discussed below, actin branching involves the formation of new actin filaments from the sides of pre-existing filaments, mediated by the Arp2/3 nucleating complex.

### Spine Shrinkage

In contrast to LTP, spine loss and shrinkage are typically observed in response to stimuli that induce long-term depression (LTD), such as low-frequency stimulation (LFS) and NMDA receptor (NMDAR) activation (Zhou et al., 2004), which induce reorganization of the actin cytoskeleton (Fischer et al., 2000; McKinney, 2010). Mechanisms of actin remodeling in LTD are not as well characterized as those that accompany LTP. However, at least for NMDA-dependent LTD, they appear to involve calcineurin/slingshot-mediated dephosphorylation (and activation) of an actin filament severing protein, cofilin (Zhou et al., 2004; Zhao et al., 2012; Figure 1), and Ca^2+^-dependent translocation of another actin filament severing protein, gelsolin, from dendritic shafts to spines (Hlushchenko and Hotulainen, 2019).

Reductions in the ratio of mature to thin spines have been linked to a variety of cognitive disorders. For example, in the case of Fragile X syndrome, there is a reduction in spine density and number and a concomitant increase in the number of thin, elongated filopodial structures (Martínez-Cerdeño, 2017). Improved microscopic imaging methods, which have allowed detailed analysis of spine shapes in postmortem tissue samples, have also revealed a higher density of filopodial spines in Alzheimer’s Disease patients than in normal aged individuals (Boros et al., 2019).

The structural plasticity of dendritic spines that accompanies learning and memory, and which goes awry in cognitive disorders, involves reorganization of the actin cytoskeleton. Proteins that govern this reorganization, and their potential regulation by palmitoylation, are described in the following sections of this review.

## RHO-Family GTPases

In an influential 1982 review, Crick (1982) hypothesized “that there are contractile proteins associated with each (dendritic) spine … which allows the spine to change its shape rapidly during neuronal activity”. Rho GTPases are the cytoskeletal regulators of dendritic shape that respond most rapidly to changes in intra-spine Ca^2+^ concentration. In humans, Rho proteins comprise a family of 18–21 (depending upon the inclusion of three “atypical” members) ~21 kDa GTPases (Woolfrey and Srivastava, 2016). Rho GTPases cycle between inactive GDP-bound and active GTP-bound states. The inactive states are stabilized by binding to GDP dissociation inhibitors (GDIs). The activation process involves dissociation of Rho proteins from their GDIs, induced in part by Src-, PKC-, or PAK-mediated phosphorylation of the GDIs, followed by GTP loading, catalyzed by GDP/GTP exchange factors (GEFs; Hodge and Ridley, 2016). Like most other members of the Ras superfamily, Rho GTPases undergo C-terminal prenylation. This irreversible modification involves the incorporation of geranylgeranyl (20-carbon) or farnesyl (15-carbon) groups on C-terminal CaaX motifs, where C is the prenylated cysteine, and aa are dual aliphatic residues, and X can be one of several amino acids. In their inactive states, GDIs mask the prenyl groups, preventing their exposure to the aqueous cytosol. The three major classes of Rho GTPases, Rho, Rac, and Cdc42, remodel the actin cytoskeleton predominantly by controlling two activities: cofilin-mediated actin severing and Arp2/3-mediated actin branching (Figure 1). Cofilin regulates the actin cytoskeleton in a complex manner (Van Troys et al., 2008). At sufficiently high concentrations of monomeric actin (G-actin), it can promote actin assembly by severing pre-existing actin filaments, thereby creating free barbed ends for the polymerization of new filaments. However, at limiting actin concentrations, cofilin-mediated severing can lead to the net loss of filamentous actin (F-actin). These diverse activities may explain why cofilin is required for both spine enlargement during LTP (Bosch et al., 2014) and spine shrinkage during LTD (Zhou et al., 2004, 2011). The Arp2/3 (actin-related proteins 2 and 3) complex binds to pre-existing actin filaments and nucleates the polymerization of new filaments at a 70° angle from the parent filaments. Thus, Arp2/3 generates branched actin networks that are required for the formation of mature, mushroom spines (Hotulainen et al., 2009). The nucleation activity of the Arp2/3 complex is triggered indirectly by Rac and Cdc42, which activate the nucleation promoting factors WAVE and WASP, respectively. As discussed below, there is strong evidence that dynamic changes in the actin network of dendritic spines are regulated by palmitoylation of Rac and Cdc42. Although palmitoylation of RhoA has been detected in multiple high-throughput screens, the effects of this modification on RhoA activity have not been explored.

## Cdc42

Since the development of two-photon glutamate uncaging (Matsuzaki et al., 2001; Kruijssen and Wierenga, 2019), it has been possible to monitor morphological changes of dendritic spines in response to induced plasticity with unprecedented spatiotemporal resolution. For example, the technique was used to demonstrate rapid and persistent enlargement of spines subjected to repeated cycles of glutamate uncaging, with corresponding increases in actin polymerization (Matsuzaki et al., 2004). Murakoshi et al. (2011) reported that upon LTP induction by glutamate uncaging, spines of CA1 pyramidal neurons in cultured rat hippocampal slices undergo a rapid, transient enlargement to about 300% of the initial volume, followed by a sustained enlargement phase (>30 min) to about 70–80% greater than initial volume. The time courses of spine enlargement and Cdc42 and RhoA activation are strongly correlated, as monitored by two-photon fluorescence lifetime imaging (FLIM) of Förster resonance energy transfer (FRET)-based sensors. RhoA is critical for the transient phase of spine enlargement, whereas Cdc42, acting through its effector, p21-activated kinase (PAK), is more strongly associated with the sustained enlargement phase, suggesting its potential importance in the maintenance of structural plasticity. Interestingly, activated Cdc42 is restricted to the stimulated spine, whereas activated RhoA and Rac1 diffuse out of the spine and spread over about 5 μm of the associated dendritic shaft. As discussed below, palmitoylation may contribute to the sequestration of a pool of Cdc42 to spines.

## Palmitoylation of Cdc42

Two splice variants of Cdc42 have been identified, differing primarily in their C-terminal membrane-binding regions (Figure 2). Both variants can be prenylated, and both contain polybasic motifs that participate in electrostatic interactions with negatively charged phospholipids. The ubiquitously expressed “canonical” variant (Cdc42^Pren^) contains a classic C-terminal CaaX prenylation motif, ^188^CVLL^191^. However, the brain-specific non-canonical variant (Cdc42^Palm^) contains a C-terminal ^188^CCIF^191^ motif, which undergoes reversible palmitoylation (Kang et al., 2008). In the mouse cerebellum, Cdc42^Pren^ is expressed at 10-fold higher levels than Cdc42^Palm^, whereas in the hippocampus the expression level of Cdc42^Palm^ is twice that of Cdc42^Pren^ (Wirth et al., 2013). Although both forms of expressed GFP-Cdc42 are found in dendrites, GFP-Cdc42^Palm^ is concentrated in spines (Kang et al., 2008). Treatment of hippocampal neurons with 50 μM glutamate for 5 min, sufficient to induce spine collapse (Halpain et al., 1998), causes a rapid depalmitoylation of Cdc42^Palm^, as well as its relocalization away from spines (Kang et al., 2008).

Prenylation of C-terminal CaaX motifs, such as ^188^CVLL^191^ of Cdc42^Pren^, is a multi-step process involving cysteine prenylation followed by proteolytic removal of the aaX residues and carboxy-methylation of the now-terminal cysteine. Whereas C^188^ of both Cdc42 variants can be prenylated, the ^188^CCIF^191^ motif of Cdc42^Palm^ suppresses post-prenylation proteolysis and carboxy-methylation. Thus, Cdc42^Palm^ may be dually lipidated, with a prenyl group on C^188^ and a palmitoyl group on C^189^ (Nishimura and Linder, 2013; Wirth et al., 2013) or with tandem palmitoyl groups on both C^188^ and C^189^ (Wirth et al., 2013).

Selective RNAi-mediated depletion of each Cdc42 variant revealed that Cdc42^Palm^, but not Cdc42^Pren^, is critical for extension of dendritic filopodia and spine formation (Kang et al., 2008). Likewise, overexpression of a constitutively active (G12V) Cdc42^Palm^ mutant had a significantly stronger spine- inducing capability in cultured hippocampal neurons than did overexpression of constitutively active Cdc42^Pren^ (Wirth et al., 2013). Because lipidation of C^188^ is essential for Cdc42 activation, expression of the C188A mutant failed to rescue the inhibitory effect of Cdc42 depletion on spine formation in hippocampal neurons.

There is indirect but strong evidence that the palmitoyltransferase ZDHHC8 is responsible for Cdc42 acylation. ZDHHC8 is particularly abundant in the brain (Fukata and Fukata, 2010) and disruption of its expression has been linked to schizophrenia (Chen et al., 2004; Mukai et al., 2004), neurodevelopmental deficits (Mukai et al., 2008), and epilepsy (Yang et al., 2018). Deletion of the 22q11.2 locus, which contains the ZDHHC8 gene, results in reduced spine density in mice (Mukai et al., 2008; Moutin et al., 2017) and a dramatic decrease in Cdc42 palmitoylation in the hippocampus (Moutin et al., 2017). Notably, spine density defects are rescued by overexpression of ZDHHC8 or a constitutively active version of Cdc42^Palm^. Cortical neurons from ZDHHC8 knockout mice also show dramatically reduced levels of Cdc42 palmitoylation, as well as impairments in axonal growth and branching (Mukai et al., 2015). Both expressed EGFP-Cdc42^Palm^ and (importantly) endogenous Cdc42^Palm^, recognized by a specific antibody, are enriched at the tips of axons. Disruption of palmitoylation, either by mutations of modified cysteines in EGFP-Cdc42^Palm^ or by treatment with the inhibitor 2-bromopalmitate, causes dispersal of Cdc42^Palm^ to the entire neuron. shRNA-mediated depletion of Cdc42^Palm^ significantly reduces axon growth and branching, as well as growth and density of dendrites, suggesting that Cdc42^Palm^ is a major target in ZDHHC8 control of neuronal structure and development. The enrichment of the palmitoylated splice variant of Cdc42 to dendritic spines and axon tips may indicate that it interacts, in a palmitoylation-dependent manner, with distinct proteins in the two locations, and may trigger distinct patterns of downstream signaling.

## Rac1

Activated Rac1 contributes to the increased actin polymerization associated with spine remodeling during LTP (Tashiro and Yuste, 2004; Rex et al., 2009). Rac1 and its downstream targets, the WAVE and Arp2/3 complexes, stimulate branched actin nucleation in the vicinity of PSDs (Chazeau et al., 2014). Using a novel biosensor to characterize Rac1 stimulation in spines, Hedrick et al. (2016) observed that Rac1 (and Cdc42) activation requires NMDAR/CaMKII-triggered postsynaptic release of BDNF followed by autocrine TrkB signaling. In contrast, NMDAR/CaMKII signaling alone (without BDNF release) is sufficient to activate RhoA in spines. In addition to being a downstream effector of CaMKII (Saneyoshi et al., 2019), Rac1 is also activated by PKCα (Tu et al., 2020). The promotion of structural LTP in hippocampal spines by PKCα requires its C-terminal PDZ-binding motif (QSAV; Colgan et al., 2018), which interacts with the PDZ domains of at least 3 palmitoylated regulators of synaptic plasticity, PICK1, PSD95, and SAP97. Using FRET-based biosensors, Tu et al. (2020) demonstrated that PKCα specifically activates Rac1, but not Cdc42 or RhoA and that the C-terminal QSAV motif is essential for Rac1 activation.

### Palmitoylation of Rac1

In response to a variety of cellular stimuli, including growth factor receptor activation and attachment to the extracellular matrix, Rac1 translocates from the cytosol to the PM, the major site of both GEF-mediated activation and GAP-mediated inactivation. In an early study, both Rac1 and RhoA were identified in caveolar rafts of quiescent fibroblasts and were found to concentrate even further in caveolae in response to PDGF treatment (Michaely et al., 1999). Subsequent work using live-cell FRET approaches and *in vitro* analysis of supported lipid bilayers gave rise to a model in which Rac1 recruitment to membranes requires cholesterol and occurs preferentially at boundaries between liquid-ordered (raft-like) and liquid-disordered (non-raft) domains (Moissoglu and Schwartz, 2014; Moissoglu et al., 2014). Following recruitment, Rac1 is free to diffuse between the raft and non-raft regions, where its activity is regulated by differential localization of GEFs and GAPs.

Stable anchoring of Rac1 to the PM requires both geranylgeranylation of cysteine 189 and the presence of an adjacent polybasic region (_183_KKRKRK_188_; Lam and Hordijk, 2013; Figure 2). Also, Rac1 can undergo palmitoylation on cysteine 178, which enhances recruitment to the PM and induces sorting to lipid rafts, as defined by incorporation into DRM fractions and colocalization in cells with the ganglioside GM1, a raft marker (Navarro-Lérida et al., 2012). Dual prenyl and palmitoyl modification of Rac1 is more conventional than that of Cdc42 because the palmitoylation event occurs outside of the CaaX box. Rac1 palmitoylation is dynamic, as it is reversed by a 30-min treatment with 2-bromopalmitate (Navarro-Lérida et al., 2012), an inhibitor that blocks palmitoylation but does not remove pre-existing palmitoyl groups. This rapid turnover may account for the relatively low levels of palmitoylation reported for Rac1. Interestingly, the largest decrease of palmitoylation in neurons from ZDHHC8 knockout mice was observed for Rac1 (38%), followed closely by Cdc42 (33%; Mukai et al., 2015).

An emerging theme in the signaling field is the existence of dynamic nanoclusters of signaling proteins in the PM (Garcia-Parajo et al., 2014; Jacobson et al., 2019), the best-characterized example being Ras, which forms approximately 16 nm complexes containing up to six Ras molecules (Zhou and Hancock, 2015). As determined by a combination of total internal reflection fluorescence (TIRF) microscopy and super-resolution single-particle tracking photoactivated localization microscopy (SPT-PALM) in fibroblasts, highly mobile Rac1 monomers were found to co-exist with ~200 nm immobile clusters containing up to 50 Rac1 molecules (Remorino et al., 2017; Maxwell et al., 2018a). The presence of Rac1 nanoclusters, which were also enriched in PIP_3_ and phosphatidic acid (PA), was confirmed by electron microscopy of PM sheets from BHK cells, although the clusters were smaller than those visualized in the super-resolution imaging study (~25 nm diameter; ~5 Rac1 molecules per cluster; Maxwell et al., 2018b). Both, nanoclustering and overall PM recruitment were inhibited by mutation of palmitoylated cysteine 178, prenylated cysteine 189, and arginine 185 within the polybasic motif, with the most profound effects being induced by impairment of prenylation. Interestingly, mutation of palmitoylated or prenylated cysteines reduced the selectivity for PIP_3_ and PA, suggesting that the lipid modifications may influence the conformational orientation of the polybasic motif.

## LIM Kinase-1 (LIMK1)

As we have seen, palmitoylation is essential for the proper localization and function of Cdc42 in dendrites and may be important in the regulation of Rac1 as well. Likewise, there is compelling evidence that palmitoylation-dependent regulation of their downstream effector, LIMK1, is critical for its ability to control cofilin activity during structural synaptic plasticity. Cofilin inactivation by phosphorylation of Ser3 is an important mechanism for the development and maintenance of the high F-actin content in dendritic spines required for LTP (Bosch et al., 2014). Cofilin-Ser3 phosphorylation is enhanced during neuronal activity (Rex et al., 2009) and is essential for activity-induced spine enlargement (Gu et al., 2010). Two important cofilin phosphorylating proteins are the LIM kinases, LIMK1 and LIMK2. We focus our consideration on LIMK1 because it is a palmitoylated protein with a well-characterized role in the regulation of actin dynamics in dendrites.

LIMK1 was first implicated in neuronal development when its deletion was identified in patients with Williams syndrome, which is associated with intellectual disability (Tassabehji et al., 1996). LIMK1 knockout mice, which display cognitive and behavioral deficiencies, have abnormal dendritic spines, with thick necks and small heads, instead of the thin necks and large heads that are typical of mature or “mushroom” spines (Meng et al., 2002). LIMK1 is activated by phosphorylation of threonine 508, catalyzed by PAKs, which are themselves activated by Rho GTPases. Double deletion of PAKs 1 and 3 in mice causes a 75% reduction in the ratio of phosphorylated (inactive) to unphosphorylated (active) cofilin, interferes with synaptic plasticity, and has major effects on spine morphology, including fewer mushroom spines, reduced head/neck ratios, and more filopodia (Huang et al., 2011).

Palmitoylation of cysteines 7 and 8 has profound effects on LIMK1 activity and localization in dendrites (George et al., 2015). First, whereas wild-type LIMK1 is highly enriched in spines, the un-palmitoylated CCSS-LIMK1 mutant is not. Both cysteines must be palmitoylated for proper sorting to spines, even if an N-terminal myristoylation motif is introduced into the single C-to-S mutants. Moreover, this dual palmitoylation motif of LIMK1 is sufficient to target GFP (and presumably other proteins) to spines. Second, CCSS-LIMK1 has a significantly higher diffusibility than wild-type LIMK1, based on fluorescence recovery after photobleaching (FRAP) data, suggesting that wild-type LIMK1 is anchored to dendritic membranes by its palmitoylation. Third, T^508^ phosphorylation of wild-type LIMK1 is about 10-fold higher than that of CCSS-LIMK1 in hippocampal neurons, suggesting that palmitoylation-dependent membrane targeting is a critical step in LIMK1 activation, likely due to the localization the (Rac/Cdc42)/PAK complex on membranes. Interestingly, wild-type LIMK1 and CCSS-LIMK1 can be phosphorylated by PAK3 at similar rates and to similar extents *in vitro*, and cofilin is phosphorylated similarly by both forms of LIMK1, indicating that the C-to-S mutations do not affect the conformation of LIMK1. Finally, knockdown of endogenous LIMK1 and replacement with the CCSS-LIMK1 mutant suppressed actin turnover in hippocampal neurons and inhibited spine enlargement induced by uncaged glutamate. Actin turnover might be expected to increase upon expression of an inactive LIMK1, as active cofilin is best characterized as an F-actin severing protein. However, at high concentrations, cofilin can bind to and stabilize (rather than sever) actin filaments (Van Troys et al., 2008). We note that cofilin concentration increases rapidly in spines during the initial phase of LTP (Bosch et al., 2014; Borovac et al., 2018).

## Arc/Arg 3.1

**Figure 3:**
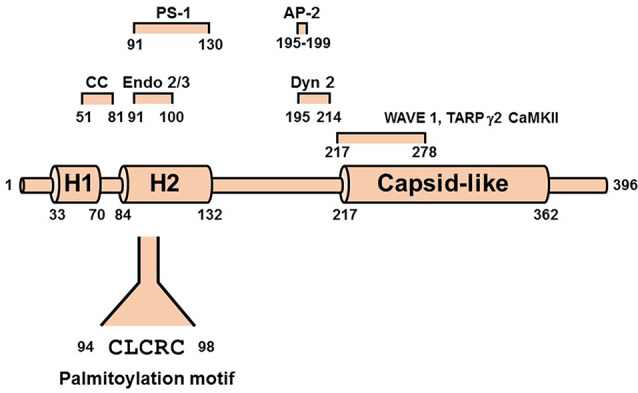
Scheme of Arc/Arg3.1 showing N-terminal helical segments H1 and H2 and the HIV capsid-like domain. Shown above the scheme are the predicted coiled-coil (CC) motif and determinants of interactions with endophilins (Endo 2/3), presenilin-1 (PS-1), clathrin adaptor protein 2 (AP-2), Dynamin 2 (Dyn2), Ca^2+^/calmodulin-dependent kinase II (CaMKII), and transmembrane AMPAR regulatory protein γ2 (TARP γ2, also known as Stargazin). The palmitoylation motif is shown below the scheme.

Arc [Activity-regulated cytoskeleton-associated protein, also known as Activity-regulated gene of 3.1 kb (Arg3.1)] is a 45 kDa immediate-early gene product encoded by an mRNA that is rapidly transcribed in response to synaptic activity (Link et al., 1995; Lyford et al., 1995). It is induced by activation of both LTP (Guzowski et al., 2000) and metabotropic glutamate receptor-dependent LTD (Park et al., 2008; Waung et al., 2008; Jakkamsetti et al., 2013), as well as by other stimuli associated with learning, memory, and behavior (reviewed in Korb and Finkbeiner, 2011; Shepherd and Bear, 2011). Arc expression is altered in a variety of cognitive disorders, including Alzheimer’s disease, Fragile-X, and Angelman’s syndromes, schizophrenia, and substance abuse (Wilkerson et al., 2018). Although Arc does not contain previously identified domains, secondary structure predictions indicate that it consists of two predominantly α-helical regions (comprising residues 1–132 and 217–396) connected by an extended disordered segment (residues 133–216; Myrum et al., 2015; Figure 3). The N-terminal region can be further divided into two helical segments, H1 (residues 33–70) and H2 (residues 84–132). Segment H2 contains a SUMOylation site (Craig et al., 2012), three predicted phosphorylation sites, and binding determinants for endophilin (Chowdhury et al., 2006) and presenilin (Wu et al., 2011). The three-dimensional structure of a C-terminal fragment (residues 217–362) was solved by x-ray crystallography and was found to be remarkably similar to that of the HIV Gag capsid domain (Zhang et al., 2015). Two studies showing that Arc assembles into capsid-like structures that enclose Arc mRNA and are released as extracellular vesicles highlight the potential physiological relevance of this structural similarity (Ashley et al., 2018; Pastuzyn et al., 2018).

**Figure 4:**
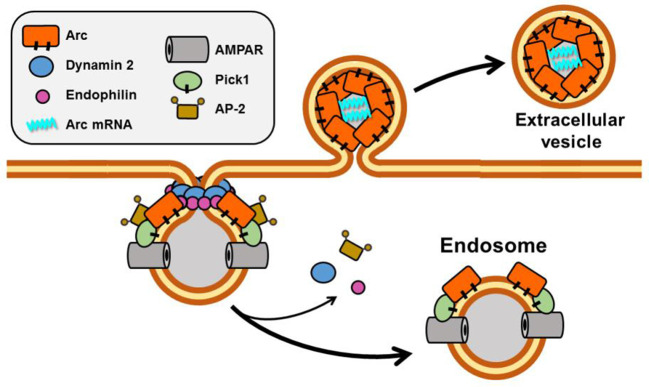
Scheme showing Arc function in membrane vesiculation. Arc interacts directly with four proteins implicated in endocytosis of AMPA receptors: Dynamin 2, Endophilin, AP-2, and PICK1. Both Arc and PICK1 are palmitoylated in neurons. Recently, Arc has also been reported to promote the release of vesicles that contain Arc mRNA.

Although the precise function of Arc in the neuron remains elusive, evidence from depletion and overexpression studies indicates that it is a positive regulator of AMPA receptor endocytosis and, hence, plays a critical role in synaptic weakening and LTD (Chowdhury et al., 2006; Rial Verde et al., 2006; Waung et al., 2008). AMPARs are internalized primarily by clathrin-mediated endocytosis (Man et al., 2000; Wang and Linden, 2000), which requires the participation of three direct Arc binding partners, dynamin, endophilin, and adaptor-protein 2 (AP-2; Chowdhury et al., 2006; Byers et al., 2015; DaSilva et al., 2016; Wall and Corrêa, 2018; Figure 4). Dynamin is a GTPase that polymerizes around the necks of invaginating vesicles and promotes GTPase-dependent membrane scission (Antonny et al., 2016). Although dynamin has been best characterized for its role in membrane vesiculation, it is becoming increasingly evident that it also functions as a regulator of actin assembly (Menon and Schafer, 2013; Sever et al., 2013). Endophilin senses and/or induces membrane curvature by its BAR (Bin- Amphiphysin-RVS) domain, which dimerizes into a crescent-shaped structure having a concave surface that binds to phospholipid bilayers. The AP-2 adaptor complex binds to receptors and to clathrin to ensure appropriate spatiotemporal regulation of coated vesicle formation.

### Arc as a Regulator of the Actin Cytoskeleton in Dendrites

As stated, there is strong evidence linking Arc expression to functional LTD. A report from the Finkbeiner lab (Peebles et al., 2010) provides a link between Arc and structural LTD. Overexpression of Arc in hippocampal cultures induces a significant increase in the ratio of thin spines and filopodia to stubby and mushroom spines. Consistent with this finding, the deletion of the Arc gene from mice has the opposite effect, increasing the proportion of mushroom to thin spines. Interestingly, overexpression of an Arc mutant lacking residues 91–100, a segment that contains both the endophilin-binding determinant and the palmitoylation motif (see below), does not affect spine morphology, whereas expression of a deletion mutant lacking the dynamin-binding determinant (residues 195–214) induces an increase in the ratio of mushroom to thin spines. The latter observation may reflect the established role of dynamin, together with the Arc-binding protein IRSp53 (insulin receptor tyrosine kinase substrate p53), in promoting the linear extension of actin filaments during filopodia formation (Chou et al., 2014).

In addition to its well-established role in synaptic weakening and LTD, Arc may also be important for synaptic strengthening and LTP (Guzowski et al., 2000; Plath et al., 2006; although see Kyrke-Smith et al., 2020 for an opposing view). Mechanisms that induce Arc to switch between these opposing processes remain somewhat enigmatic (Tzingounis and Nicoll, 2006). However, Arc’s role in synaptic strengthening has been most convincingly linked to its activity as a regulator of the actin cytoskeleton (Bramham, 2008). Indeed, Arc was first identified as a protein that associates with the actin cytoskeleton, although it does not bind directly to actin (Lyford et al., 1995). An early study linking reduced Arc expression with suppression of LTP demonstrated that Arc promotes inactivation of cofilin by inducing its phosphorylation on serine 3 (Messaoudi et al., 2007).

How can Arc promote both actin polymerization, which is associated with spine enlargement, and actin disassembly, which is associated with spine shrinkage? A recent attempt to resolve this paradox postulates a shift in Arc binding partners triggered by Arc SUMOylation and deSUMOylation (Nair et al., 2017). SUMOylation, which is activated in response to LTP-inducing stimuli, promotes interaction between Arc and the actin-stabilizing protein, drebrin. In contrast, deSUMOylation disrupts the Arc-drebrin interaction, freeing Arc to activate cofilin.

### Palmitoylation of Arc

As shown in our recent study (Barylko et al., 2018), Arc undergoes palmitoylation in a cysteine-rich motif (_94_CLCRC_98_) within the helical segment H2 (Figure 3). Because Arc is found predominantly in detergent-insoluble complexes in the brain (Lyford et al., 1995) and synaptosomes (Barylko et al., 2018), we considered the possibility that palmitoylation drives Arc into detergent-resistant subcellular compartments. Synaptosomes contain two major detergent-insoluble fractions, PSDs, and lipid rafts (Suzuki, 2002). Approximately half of the detergent-insoluble portion of synaptosomal Arc co- fractionates with low buoyant density rafts in sucrose density gradients, although only about 10% of synaptosomal Arc is estimated to be palmitoylated. This discrepancy may be explained by the putative direct or indirect interaction of Arc with PSD95, which is anchored to raft membranes *via* two palmitoylated cysteines in its N-terminus. Arc has also been reported to facilitate the proteolytic processing of amyloid precursor protein (APP) by γ-secretase on intracellular vesicles (Wu et al., 2011), which is believed to occur on rafts (Bhattacharyya et al., 2013). The precise functions of Arc palmitoylation in regulating synaptic plasticity remain to be defined. However, the expression of a non-palmitoylatable mutant in Arc KO mice failed to rescue Arc-dependent mechanisms of synaptic depression. In a prior investigation, Arc was found to be essential for synapse depression induced by expression of MEF2-VP16, a constitutively active form of the activity-dependent transcription factor, MEF2 (myocyte enhancer factor 2; Wilkerson et al., 2014). Co-expression of wild-type Arc and MEF2-VP16 rescues MEF2-induced synapse elimination in Arc knockout neurons. However, a non-palmitoylatable Arc mutant, Arc^C94,96,98S^, was unable to rescue the MEF2 effect (Barylko et al., 2018), indicating that palmitoylation is necessary for MEF2-induced synapse elimination.

## PICK1 (Protein Interacting with C-Kinase 1)

**Figure 5:**
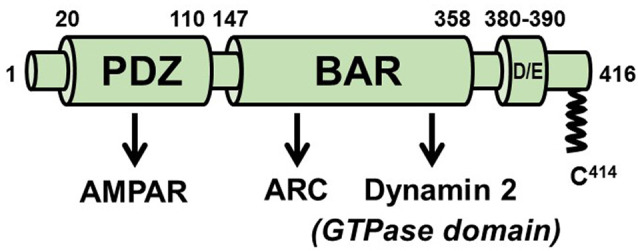
Scheme of PICK1. The PDZ domain mediates numerous functional PICK1-protein interactions, including with the AMPA receptor. The BAR domain is required for the association of PICK1 with membranes but also engages in protein interactions with Arc and with the GTPase domain of dynamin 2. Approximately 5–10% of PICK1 is palmitoylated on cysteine 414.

PICK1 contains an N-terminal PDZ (PSD95/*discs large*/ZO-1) domain that binds directly to the C-termini of GluA2 and GluA3 AMPAR subunits, a BAR domain that binds lipids and senses and/or generates membrane curvature, and a C-terminal acidic region (Figure 5). Many studies have implicated PICK1 in LTD (e.g., Steinberg et al., 2006; Terashima et al., 2008; Volk et al., 2010). Its association with Rab11-positive endosomes and inhibition of their recycling to the PM (Madsen et al., 2012), lends support to models suggesting that PICK1 contributes to synaptic weakening by inhibiting AMPAR recycling rather than its internalization from the PM (Steinberg et al., 2006; Lin and Huganir, 2007; Citri et al., 2010; Widagdo et al., 2016). However, a more recent study (Fiuza et al., 2017; Hanley, 2018) using stimulated emission depletion (STED) super-resolution imaging demonstrated colocalization of GFP-PICK1 with clathrin clusters, a subset of which were distributed to the endocytic zones (EZs; Blanpied et al., 2002) adjacent to PSDs. It was further shown that, like Arc, PICK1 binds directly to both AP-2 and dynamin 2. The PICK1-AP-2 interaction is mediated by two DVF motifs in PICK1, one in the BAR domain and the other immediately C-terminal to the BAR domain. The PICK1-dynamin interaction also occurs *via* the BAR domain of PICK1 which, almost unique among dynamin-binding proteins, associates with the dynamin GTPase domain. Thus, it appears that PICK1 controls multiple stages in the AMPAR endocytic pathway.

PICK1 was also proposed to weaken synaptic strength and induce spine shrinkage by promoting actin disassembly. According to this mechanism, PICK1 competitively inhibits the interaction between Arp2/3 and N-WASP *via* binding of its BAR domain to actin and its C-terminal acidic region to the actin-nucleating Arp2/3 complex (Rocca et al., 2008; Nakamura et al., 2011). However, conflicting evidence indicating that PICK1 neither binds to Arp2/3 nor inhibits Arp2/3-mediated actin nucleation was also reported (Madasu et al., 2015). The basis of this discrepancy is unclear.

### Palmitoylation of PICK1

Although PICK1 is a soluble protein, it can associate with membranes *via* direct binding to lipids of its PDZ domain (Pan et al., 2007), BAR domain (Jin et al., 2006), and an amphipathic helix in the ~40 amino acid linker that connects these two domains (Erlendsson and Madsen, 2015). A relatively small pool of PICK1 is also tethered into the hydrophobic core of the bilayer by palmitoylation, primarily on cysteine 414 (C^414^) near the C-terminus (Thomas et al., 2013). The palmitoyltransferase responsible for PICK1 palmitoylation is ZDHHC8, which contains a C-terminal PDZ domain-binding motif required for PICK1 binding. Interestingly, ZDHHC8 knockdown in mice results in decreased surface expression, but increased cytoplasmic expression, of GluA1 subunits, without overall changes in total GluA1 levels (Yang et al., 2018). Thus, ZDHHC8 may promote decreased endocytosis and/or increased recycling of GluA1, whereas PICK1 promotes increased endocytosis and/or decreased recycling of GluA2. Induction of cerebellar LTD is suppressed upon mutation of C^414^ of PICK1 to serine and by knockdown of ZDHHC8 (Thomas et al., 2013).

### Interaction Between PICK1 and Arc

Although PICK1 and Arc have both been implicated in promoting AMPAR clearance from the PM, and both proteins bind to dynamin and AP-2, a direct interaction between them was only reported in late 2018 (Goo et al., 2018). In this report, a combination of biochemical and fluorescence-based nanoimaging approaches was used to detect Arc-PICK1 complexes *in vitro* and the cytosol and PM of live SH-SY5Y cells. Interactions were enhanced upon membrane depolarization, suggesting responsiveness to neuronal activation. The Arc binding determinant was localized to residues 152–278 of PICK1, within the N-terminal region of the BAR domain. The PICK1-binding determinant within Arc has not yet been identified. It is unlikely that palmitoylation functions by localizing PICK1 to lipid rafts, as cerebellar LTD can be rescued in ZDHHC8-depleted neurons by replacing the palmitoylated cysteine (C^414^) in PICK1 with a C-terminal prenylation (CaaX) motif (Thomas et al., 2013), a modification that segregates proteins to non- raft domains (Levental et al., 2010). However, one may speculate that the palmitoylation of PICK1 serves to strengthen its association with the membrane-associated pool of Arc.

## Tetraspanins

Tetraspanins comprise a family of 33 (in mammals) structurally related proteins that play a role in cell adhesion, migration, and proliferation (Hemler, 2005). They assemble into hetero-oligomers and recruit other trans-membrane proteins to form supramolecular complexes on the PM known as “tetraspanin webs” or “tetraspanin-enriched microdomains” (TEMs). Palmitoylation of tetraspanins is essential for their incorporation into TEMs (Termini and Gillette, 2017). A common feature of several tetraspanins is their association with integrins (Berditchevski, 2001; Bassani et al., 2012) and with the palmitoylated Type II phosphatidylinositol 4-kinases (PI4KIIs; Yauch and Hemler, 2000).

### Tetraspanin 7 (TSPAN7)

**Figure 6:**
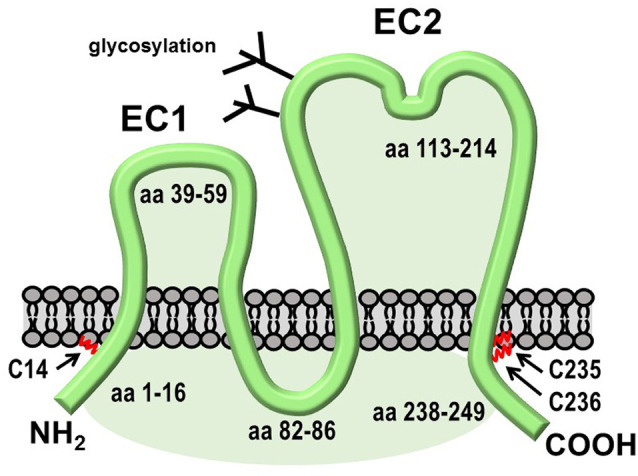
Structural features of TSPAN7. Like other tetraspanins, TSPAN7 contains four transmembrane domains, one large and one small extracellular region, and three small cytoplasmic segments. The C-terminal segment contains a PDZ-binding motif that interacts with PICK1.

TSPAN7 (also known as Cell Surface Glycoprotein A15 or TALLA1) is a 249 amino acid protein that is most highly expressed in the brain. Palmitoylation of TSPAN7, which was directly demonstrated by incorporation of radioactive palmitate (Yang et al., 2002), is predicted to occur on both N- and C-terminal cytoplasmic segments, according to the GPS-Lipid program (Xie et al., 2016; Figure 6). Mutations in the *TM4SF2* gene, which encodes TSPAN7, have been linked to human cognitive disorders (Zemni et al., 2000). Synaptic phenotypes resulting from TSPAN7 overexpression or knockdown resemble those observed upon the manipulation of Arc expression (Bassani et al., 2012). TSPAN7 overexpression in cultured rat hippocampal neurons induces the formation of filopodia and spines, whereas its depletion causes an increase in the proportion of thin vs. mushroom-shaped spine heads, prevents LTP-induced spine enlargement and increased spine density, and decreases AMPAR current and AMPAR surface expression. Mechanisms underlying these phenotypes may involve recruitment by TSPAN7 of PICK1 and β1 integrins into TEMS on the postsynaptic surface (Bassani and Passafaro, 2012). The cytoplasmic tail of TSPAN7 binds directly to the PDZ domain of PICK1, apparently competing for PICK1 binding to GluA2 subunits of AMPARs. Thus, the depletion of TSPAN7 may promote synaptic weakening by allowing an increase in PICK1- mediated clearance of AMPARs from the dendritic PM. More recently, the deletion of the tetraspanin protein TSPAN6 was identified in patients with early infantile epileptic encephalopathy-9 (EIEE9), but a role for TSPAN6 in spine remodeling could not be established (Salas et al., 2017).

Insight into the mechanism of actin regulation by TSPAN7 was provided by a study aimed at explaining TSPAN7 involvement in HIV-1 transfer from dendritic cells of the innate immune system to T cells (Ménager, 2017). Depletion of TSPAN7 from dendritic cells by shRNA resulted in the conversion of long, thin surface protrusions (“dendrites”) to short blebs which, notably, were devoid of actin filaments. A similar conversion occurred upon the introduction of drugs that inhibit the Arp2/3 complex, raising the possibility that TSPAN7 is a positive regulator of Arp2/3-mediated actin nucleation. If this mechanism proves to be correct, it raises the possibility that the TSPAN7 inhibits both PICK1-dependent AMPAR endocytosis and PICK1-mediated inhibition of Arp2/3.

### Tetraspanin 5 (TSPAN5)

Recent work from Moretto et al. (2019) has demonstrated the significance of TSPAN5 in spine maturation. TSPAN5 is present during nearly every stage of spine maturation, beginning with filopodia, but seems to function primarily in the conversion of immature spines to mushroom spines. Knockdown of TSPAN5 results in a decrease in spine density and a striking reduction in the ratio of mushroom to thin spines. This effect is mediated, at least in part, by TSPAN5-dependent clustering of neuroligin 1 (NLG1), to which it interacts *via* its large extracellular loop. The depletion of TSPAN5 also increases the lateral mobility of NLG1 on the postsynaptic membrane, consistent with a role in incorporating NLG1 into TEMs. Palmitoylation of TSPAN5, which has been directly demonstrated (Eschenbrenner et al., 2019), is not required for NLG1 clustering plays a role in reducing the lateral mobility of NLG1. This finding suggests that high-order TSPAN5 interactions, which are dependent upon palmitoylation, may stabilize the TSPAN5/NLG1 microdomain.

## Conclusion and Perspective

The focus of this review has been on palmitoylated proteins directly implicated in remodeling of the actin cytoskeleton during synaptic plasticity, whether or not the functional significance of their palmitoylation has been conclusively established. Several other palmitoylated proteins that function in other capacities in the dendritic spine (e.g., in the organization of the PSD or maintenance of cell adhesions) also have a critical, albeit less direct, impact on structural synaptic plasticity. For example, Chazeau et al. (2014) used single-particle tracking and super-resolution imaging to show that the PSD itself is a key organizing center for the nucleation of actin branching. In contrast to neuronal growth cones or fibroblast lamellipodia, where regulators of actin branching and elongation are co-distributed to the protruding membrane, these complexes are spatially separated in spines. Notably, elements of the branching machinery, including IRSp53, WAVE, and Ap2/3, are largely immobilized on the spine plasma membrane adjoining the PSD, whereas actin filament elongation proteins [e.g., VASP and formin-like protein 2 (Fmnl2)] are concentrated at the tips of thin projections that extend away from the PSD. The association of this actin nucleating complex with the PSD may be mediated in part by direct interactions between PSD95 and IRSp53 (Choi et al., 2005).

A palmitoylated protein that functions primarily in the regulation of synaptic adherens junctions but may also influence actin assembly is δ-catenin (Kosik et al., 2005). Catenins comprise a family of proteins that interact with cytoplasmic domains of cadherins, transmembrane proteins that mediate the formation of cell-cell junctions. The best-characterized family member is β-catenin, which functions both as an adhesion molecule and a transcription factor. Although palmitoylation of β-catenin has been detected in multiple high-throughput screens and at least one directed study (Stypulkowski et al., 2018), palmitoylation of δ-catenin has been more extensively investigated, particularly in the context of synaptic plasticity. All members of the p120 catenin sub-family, of which δ-catenin is a member, have been implicated in the regulation of Rho family GTPases (Anastasiadis, 2007). p120 itself has been shown by several groups to activate Rac1 and Cdc42 while inhibiting RhoA. Consistent with these findings, overexpression of δ-catenin in lymphatic endothelial cells activates Rac1 and Cdc42 but inhibits RhoA (Ghose et al., 2015). The knockdown of δ-catenin had opposite effects. The mechanism of δ-catenin-mediated regulation of Rho GTPases is unclear, but there is biochemical evidence based on experiments with purified proteins that p120 binds directly to RhoA and inhibits GDP/GTP exchange, acting in effect as a GDI (Anastasiadis et al., 2000). Activation of Rac1 and Cdc42 may instead be the result of RhoGEF activation by p120 family catenins (Noren et al., 2000).

δ-Catenin is palmitoylated by zDHHC5, which is sensitive to synaptic activity. Upon LTP-inducing stimuli, zDHHC5 dissociates from PSD95, undergoes endocytosis, and travels on recycling endosomes into the dendritic shaft where it encounters and modifies δ-catenin (Brigidi et al., 2015). Acylated δ-catenin is then recruited to the dendritic spine, where it forms a complex with N-cadherin and AMPAR. It is currently unknown how palmitoylation influences the ability of δ-catenin to regulate Rho GTPases. Because Rho GTPase activation and inhibition occur predominantly on membranes, it may be expected that palmitoylation enhances the regulatory activity of δ-catenin.

**Figure 7:**
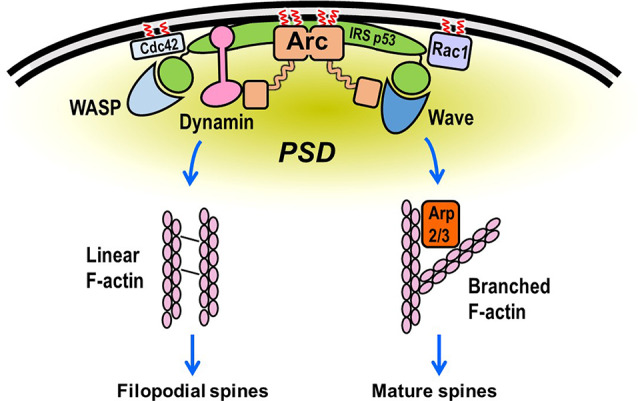
Hypothetical scheme showing assembly of the actin regulatory machinery in the dendritic spine. Rac and Cdc42 bind to the plasma membrane (PM) *via* C-terminal prenylation and, in some cases, palmitoylation. They then recruit the WAVE and WASP complexes to the PM, where they stimulate Arp2/3-mediated actin filament branching from the PSD. Rac activation of WAVE is facilitated by IRSp53, which binds to membranes directly but is not known to undergo lipidation. Cdc42 can also bind to IRSp53, promoting the formation of a Cdc42/IRSp53/VASP complex. This complex induces actin filament elongation (by VASP) and membrane deformation (by IRSp53; Disanza et al., 2013), perhaps resulting in the formation of filopodia-like extensions from regions adjacent to the PSD (Chazeau et al., 2014). Arc, which is preferentially targeted to lipid rafts by palmitoylation, binds to both WAVE and IRSp53, which may partially account for its reported participation in both long-term potentiation (LTP) and spine enlargement and long-term depression (LTD) and spine shrinkage. Although this model is highly speculative, it depicts many of the interactions described in this review.

The idealized scheme in Figure 7 depicts many of the interactions discussed above. Although each binary interaction shown in the scheme has been validated, it is not clear how many of the interactions occur simultaneously. Both actin branching and network formation are likely to involve IRSp53, which binds, directly or indirectly, to several of the proteins discussed in this review. For example, IRSp53 facilitates the interaction of Rac with the WAVE complex. Moreover, whereas Arc itself is not known to interact directly with either Rho GTPases or their downstream kinases, it may bind indirectly *via* its established interaction with IRSp53 (Purcell et al., 2014). Although IRSp53 is not known or predicted to be palmitoylated, it is likely to sort to PSD rafts by its interaction with PSD95. The model presented in Figure 7 does not address the paradoxical findings that Arc promotes both spine enlargement (Messaoudi et al., 2007) and induction of thin, filopodia-like spines (Peebles et al., 2010). However, it should be noted that IRSp53 has been implicated in both Rac1/WAVE/Arp2/3-mediated actin branching and in Cdc42/VASP-mediated filopodia formation (Krugmann et al., 2001). Possibly the signaling mechanisms accompanying synaptic strengthening and weakening induces distinct patterns of post-translational modifications of actin regulatory proteins. These modifications, such as SUMOylation of Arc mentioned above, may cause these proteins to switch between the promotion of actin branching and linear actin elongation.

Several proteins that regulate both the actin cytoskeleton and dendrite morphology, including the ArpC3 subunit of Arp2/3, the WAVE component Cyfip1, and the pseudokinase CamKv, have been identified in multiple screens for palmitoylated proteins^1^. Future studies aimed at establishing the validity of these modifications and assessing their significance promise to resolve current outstanding questions and provide insights into the role of palmitoylation in the regulation of actin-mediated remodeling of dendritic spines.

## Author Contributions

All authors participated in all stages of the preparation of this manuscript.

## Conflict of Interest

The authors declare that the research was conducted in the absence of any commercial or financial relationships that could be construed as a potential conflict of interest.

## Footnotes

^1^https://swisspalm.org
